# Photodynamic Therapy with Natural Photosensitizers: Bridging Oncology, Infectious Diseases, and Global Health

**DOI:** 10.3390/pharmaceutics17121551

**Published:** 2025-12-01

**Authors:** Renato Sonchini Gonçalves, Gustavo Braga

**Affiliations:** 1 Laboratory of Chemistry of Natural Products, Department of Chemistry, Federal University of Maranhão (UFMA), São Luís 65080-805, Brazil; 2 University College (COLUN), Federal University of Maranhã (UFMA), São Luís 65080-805, Brazil; gustavo.braga@ufma.br

## 1. Introduction

Photodynamic therapy (PDT) has undergone a remarkable transformation since its inception more than a century ago, evolving from a niche experimental technique into a versatile biomedical platform with wide-ranging therapeutic applications [1,2]. At its core, PDT relies on three interconnected components: a photosensitizer, a light source, and molecular oxygen to generate reactive oxygen species (ROS) that exert cytotoxic, antimicrobial, or immunomodulatory effects [3,4,5]. What distinguishes PDT from many conventional therapies is its spatio-temporal selectivity, the possibility of repeated administration without cumulative toxicity, and its ability to act through multimodal mechanisms that include direct cell death, vascular damage, and immune system activation [6,7,8,9].

In recent decades, the refinement of light sources, the discovery of novel photosensitizers, and the advent of nanotechnology have considerably expanded the therapeutic window of PDT [10,11,12]. Parallel advances in molecular and cellular biology have revealed that PDT is not only a destructive therapy, but also a powerful modulator of tumor microenvironments, microbial biofilms, and host immune responses [13,14,15]. In this context, natural photosensitizers have gained attention as sustainable and biocompatible alternatives to synthetic compounds, offering lower toxicity, chemical diversity, and alignment with green chemistry principles. Identification of natural photosensitizers such as hypericin, riboflavin, curcumin, and anthraquinones has further aligned PDT with the principles of sustainability and green chemistry, while simultaneously broadening its chemical and mechanistic diversity [16,17,18,19,20].

These innovations coincide with urgent global health needs. In oncology, PDT provides alternatives for tumors resistant to conventional therapies [21]; in infectious diseases, it emerges as a strategy against antimicrobial resistance [22,23]; and in dentistry and dermatology, it offers minimally invasive approaches for biofilm control, wound healing, and periimplantitis [24]. In particular, PDT shows promise for neglected tropical diseases such as leishmaniasis, where accessible treatments remain scarce [25,26].

This Special Issue of *Pharmaceutics* reflects these advances through studies on molecular mechanisms, translational research, nanotechnology, and systematic reviews. Collectively, the contributions underscore the cytotoxic, immunomodulatory, and antimicrobial potential of PDT, strengthening its evolution into an integrative, patient-centered modality. By situating PDT at the intersection of chemistry, nanotechnology, immunology, and clinical science, this Special Issue demonstrates a paradigm shift. PDT is no longer a peripheral drug, but a rapidly maturing therapeutic strategy to tackle pressing biomedical challenges.

## 2. Overview of Published Work

The articles collected in this Special Issue provide a broad and multifaceted perspective on the potential of PDT, encompassing mechanistic, translational, and clinical viewpoints.

At the cellular and molecular level, Olek et al. provided compelling evidence for the immunomodulatory effects of hypericin-mediated PDT in oral cancer cells [18]. Their findings demonstrate not only direct cytotoxicity but also the activation of immune-related pathways, suggesting that PDT can act as a bridge between local tumor ablation and systemic immune engagement. Extending these observations, Krupka-Olek et al. confirmed the dual cytotoxic and immunomodulatory role of hypericin in skin cell cultures, reinforcing the relevance of this natural compound as a clinically translatable photosensitizer for dermatological malignancies [27].

In the context of infectious diseases, Jeong and Hwang revealed that natural phytochemicals combined with visible light irradiation display a potent synergistic antibacterial activity against *Staphylococcus aureus* [21]. This contribution is particularly relevant in the era of antimicrobial resistance, as it demonstrates that PDT can bypass classical resistance mechanisms and expand the therapeutic arsenal against multidrug-resistant pathogens. In addition, Marioni et al. highlighted the antifungal potential of natural anthraquinone parietin, showing its ability to disrupt Candida tropicalis biofilms through photodynamic mechanisms [23]. These results broaden the chemical diversity of natural photosensitizers and support the applicability of PDT in fungal infections, a field traditionally limited in therapeutic options.

Three systematic reviews strengthened the translational scope of this Special Issue. Łopaciński, et al. analyzed riboflavin- and hypericin-mediated PDT for oral candidiasis, integrating preclinical and clinical data to demonstrate promising efficacy in the management of opportunistic fungal infections [28]. Fiegler-Rudol et al. evaluated the role of riboflavin-mediated PDT in periodontology, critically assessing its benefits for periodontal regeneration and biofilm control [20]. Warakomska et al. extended these insights to periimplantitis, systematically demonstrating how PDT, mediated by natural photosensitizers, can reduce microbial burden and inflammation while preserving implant integrity [24]. Together, these reviews underscore the consolidation of PDT as an evidence-based adjunct in oral medicine and dentistry.

From a nanotechnological and translational point of view, Campos et al. introduced an innovative nanogel formulation coencapsulating curcumin and essential oil *Pectis brevipedunculata*, designed for daylight-mediated PDT against leishmaniasis [25]. This study exemplifies how nanostructured delivery systems can enhance the solubility, stability, and synergistic activity of natural products, while addressing neglected tropical diseases that disproportionately affect low-resource regions. Beyond its biomedical implications, the work also highlights the importance of integrating sustainability and accessibility in the innovation of PDT.

Taken together, the contributions in this Special Issue highlight the breadth and dynamism of PDT research. They collectively reveal how natural products, nanotechnology, and systematic clinical evaluation are converging to redefine PDT as a clinically relevant, sustainable, and patient-centered therapeutic strategy. Table 1 provides a consolidated overview of the natural photosensitizers discussed in the articles published in this Special Issue, summarizing their biological sources, irradiation parameters, and therapeutic applications. It is not intended as an exhaustive list of all photosensitizers employed in photodynamic therapy.

## 3. Future Perspectives

The insights from this Special Issue highlight both the remarkable achievements and the persisting gaps that will shape the trajectory of photodynamic therapy (PDT) in the coming decade. Collectively, the contributions emphasize that PDT is transitioning from an experimental or adjunctive technique to a mainstream therapeutic strategy, supported by strong preclinical evidence, growing clinical validation, and innovative technological platforms. Nevertheless, for PDT to achieve its full translational and societal impact, several priorities must be pursued.

1. Clinical Standardization and Protocol Harmonization: The field urgently requires harmonized preclinical and clinical protocols to improve reproducibility and facilitate regulatory approval. Establishing standardized parameters for light dosimetry, photosensitizer concentration, and treatment regimens will enhance cross-study comparability and accelerate clinical translation.
2. Nano- and Bioengineered Photosensitizer Platforms: Advances in nanotechnology—such as stimuli-responsive nanogels, hybrid lipid-polymeric carriers, and bioinspired delivery systems—should be leveraged to improve selectivity, tissue penetration, and pharmacokinetics. Theranostic nanoplatforms capable of combining imaging, controlled release, and therapeutic action represent a frontier for precision PDT.
3. Integration with Immunotherapy and Host Modulation: PDT’s ability to induce immunogenic cell death and release danger-associated molecular patterns (DAMPs) positions it as a synergistic partner to immune checkpoint inhibitors and vaccines. Exploring PDT as a bridge between local ablation and systemic immunity could redefine its role in oncology and infectious disease management.
4. Applications in Neglected and Emerging Diseases: The promising results against Leishmania parasites and fungal biofilms point to the potential of PDT in neglected tropical diseases and hard-to-treat infections. Future research should emphasize low-cost, daylight-mediated PDT approaches adaptable to resource-limited settings, reinforcing the alignment with global health equity.
5. Green Chemistry and Sustainable Development: The increasing reliance on natural products as photosensitizers highlights the relevance of sustainability. Bioprospecting guided by ethnopharmacology, combined with scalable green extraction and synthetic biology approaches, can ensure eco-friendly and socially responsible production of next-generation photosensitizers.
6. Artificial Intelligence and Predictive Modeling: The incorporation of artificial intelligence and systems biology into PDT research holds promise for optimizing treatment planning, predicting patient-specific responses, and designing adaptive protocols. These tools can also accelerate the rational design of novel photosensitizers with improved photophysical and pharmacological profiles.

## Figures and Tables

**Table 1:** Summary of natural photosensitizers included in the articles published in this Special Issue, their biological sources, irradiation parameters, and therapeutic applications in photodynamic therapy (PDT).

Photosensitizers (Concentration)	Natural Source	Wavelength Range (nm)	Application/Carrier Medium
Hypericin (0.25–1.0 µM)	*Hypericum perforatum*	580–720	Oral carcinoma/DMSO solution
Immunomodulation; cytotoxicity [18].			
Hypericin (0.1–1.0 µM)	*Hypericum perforatum*	450–720	Inflammatory dermatoses/DMSO solution
Reduced inflammation; apoptosis [27].			
Plant extracts (31–500 µg/mL)	*Sophora officinalis*, *Ulmus davidiana*, *Cimicifuga simplex*, *Glycyrrhiza uralensis*	465–625	Antibacterial therapy/Aqueous extract
Synergistic antibacterial effect [21].			
Parietin (0.98 µg/mL)	*Teloschistes nodulifer*	428	Antifungal therapy/Hydroalcoholic sol. (1%)
Biofilm disruption via ROS [23].			
Riboflavin and Hypericin (0.25–320 µM)	*Aspergillus gossypii*, *Hypericum perforatum*	365–750	Oral candidiasis/Micelles (P123)–lipid carriers
Biofilm inactivation; antifungal synergy [28].			
Riboflavin (10–100 µg/mL)	*Aspergillus gossypii*, *Bacillus subtilis*, *Arabidopsis thaliana*	390–670	Periodontology/Hydrogel–NP dispersion
Adjunct antimicrobial action [20].			
Curcumin, Riboflavin, 5-ALA, Hypericin (0.1–0.5%)	*Curcuma longa*, *Aspergillus gossypii*, *Bacillus subtilis*, *Hypericum perforatum*	390–810	Peri-implantitis/ Nanogels–biopolymers
Reduced microbial load and inflammation [24].			
Curcumin (2–18 µg/mL)	*Curcuma longa* and *Pectis brevipedunculata*	Daylight	Antileishmanial therapy/Pluronic F127–Carbopol 974P
Anti-*Leishmania* effect; synergistic PDT [25].			
